# “On‐Water” Interfacial Acidification Enhances Direct Ammonolysis of Triglycerides

**DOI:** 10.1002/cssc.202500912

**Published:** 2025-08-12

**Authors:** Jie Gao, Kang Wang, Yunjiao Gu, Marc Pera‐Titus

**Affiliations:** ^1^ Eco‐Efficient Products and Processes Laboratory (E2P2L) UMI 3464 CNRS‐Solvay Shanghai 201108 China; ^2^ Cardiff School of Chemistry Cardiff Catalysis Institute Translational Research Hub Cardiff University Maindy Road Cardiff CF24 4HQ UK; ^3^ Solvay China (Co) Shanghai 201108 China

**Keywords:** Aquivion PFSA, hydrolysis, interfacial catalysis, pickering emulsion, triglyceride

## Abstract

Direct ammonolysis of triglycerides with liquid ammonia is a well‐established technology for the industrial manufacture of aliphatic amides typically requiring a large excess of ammonia. In this study, using glyceryl trilaurate (GTL) as a model triglyceride, we demonstrate that the rate of lauramide formation can be enhanced sevenfold at 150 °C and low ammonia excess (47:1 mol/mol ammonia/GTL) when the reaction is carried out in GTL‐in‐water(acetonitrile) (50:50 v/v) emulsions stabilized by surface‐active Aquivion perfluorosulfonic superacid (PFSA) particles, compared to a non‐emulsified system. Moreover, the lauramide yield after 5 h is nine times higher than that achieved with conventional ammonolysis in liquid ammonia at higher ammonia excess (148:1 mol/mol ammonia/GTL). This significant increase in activity is attributed to the “on‐water” selective acidification at the microstructured oil–water interface facilitated by adsorbed Aquivion PFSA particles, in combination with enhanced ammonia solubilization and selective glycerol extraction by the water(acetonitrile) phase.

## Graphical abstract

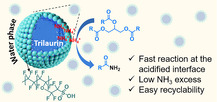

## 1 Introduction

Fatty amides are key ingredients in the chemical industry, used in the production of polymers, surfactants, lubricants, antiblocking agents, corrosion inhibitors, pigment dispersants, biopolymer‐based paints and surface coatings.^[^ ^1^ ^]^ Industrially, fatty amides are synthesized via the ammonolysis of triglycerides or fatty acids at elevated temperatures (>200 °C), under high ammonia (NH_3_) excess, and autogenous pressure typically exceeding 100 bar to maintain NH_3_ in the liquid phase.^[^ ^2^ ^]^

Ammonolysis of esters is known to proceed through an acid‐base mechanism autocatalyzed by NH_3_, with the rate of ammonolysis increasing alongside the acid strength of the alcohol leaving group,^[^ ^3^ ^]^ and the polarity of the solvent (e.g., water, ethylene glycol, glycerol, and acetonitrile (ACN)).^[^ ^4^ ^]^ The rate‐limiting step is attributed to the formation of an amino‐alcoholate, six‐membered ring, zwitterionic tetrahedral transition state stabilized by two NH_3_ molecules, with minimal cleavage of the C‐OR’ bond (R’ = aliphatic, or aromatic group).^[^ ^3^ ^]^ Catalysis using ammonium salts,^[^ ^5^ ^]^ enzymes (e.g., lipases),^[^ ^6^ ^]^ and solids acids (e.g., boron phosphate, HBEA‐75, Pt/ZrO_2_, and Al_2_O_3_)^[^ ^7^ ^]^ has been shown to accelerate ammonolysis of esters and triglycerides (e.g., soybean oil) by promoting protonation of the R’O leaving group within the zwitterionic transition state^[^ ^8^ ^]^ and may also enable alternative pathways involving noncyclic transition states.

Surfactant micelles and microemulsions have been reported to accelerate the ammonolysis/aminolysis of esters via “on‐water” effects. Griffin et al. demonstrated that micelles formed from perfluorononanamide (PFNA) in liquid NH_3_ catalyzed the ammonolysis of propargyl benzoate with a 14‐fold rate enhancement at PFNA concentrations above the critical micelle concentration (42 mM).^[^ ^9^ ^]^ This rate increase was attributed to stabilization of the zwitterionic transition state at the micelle‐NH_3_ interface due to the high interfacial polarity and H‐bonding. Similarly, Mirgorodskaya et al. reported a twofold increase in the second‐order rate constants for the aminolysis of *p*‐nitrophenyl acetate (NPA) with octylamine in water‐in‐oil (inverse) emulsions stabilized by cetyltrimethylammonium (CTAB) bromide, relative to oil‐in‐water (direct) emulsions, owing to a higher reactant concentration at the interface.^[^ ^10^ ^]^ Garcia‐Rio et al. observed comparable effects for the aminolysis of NPA with morpholine in water‐in‐oil emulsions (isooctane as oil phase) stabilized by Aerosol OT.^[^ ^11^ ^]^ In another example, Ragupathy et al. achieved substantially higher amide yields in the lipase‐catalyzed aminolysis of lactones (e.g., pentadecanolide) with aliphatic amines (e.g., oleylamine) in oil‐in‐water miniemulsions stabilized by the nonionic surfactant Lutensol AT50, compared to aqueous conditions (>90% vs. 7%).^[^ ^12^ ^]^ Kinetic studies revealed that the lipase first catalyzed lactone hydrolysis, followed by amidation.

Of particular relevance to “on‐water” catalysis is the acid‐base character of the water‐oil (and air‐water) interface, which remains a subject of debate in the literature (see ref. [13] and references therein). Water at interfaces can exhibit greater self‐ionization than in the bulk, and interfacial H_3_O^+^ and HO^−^ species can act as a stronger acid and base, respectively, than their bulk counterparts.^[^ ^14^ ^]^ This behavior has been attributed to limited hydration at the interface and intense local electric‐field gradients.^[^ ^15^ ^]^ Moreover, the interfacial dissociation of inorganic acids such as HCl and HNO_3_ can proceed more rapidly than in bulk water, although the presence of adsorbed ions may influence this effect.^[^ ^16^ ^]^

Herein, we designed a particle‐stabilized (Pickering) oil‐in‐water emulsion system to investigate whether interfacial acidification can drive enhanced ammonolysis of vegetable oils, offering a more sustainable route to amide production. We employed Aquivion Perfluorosulfonic Superacid (PFSA, PW98, 1.02 mmolH^+^/g), formulated as particles and displaying surface‐active properties,^[^ ^14^ ^]^ to achieve simultaneous emulsification and acid catalysis at the microstructured oil‐water interface. The effect of co‐solvents (e.g., acetonitrile, ethanol) was also examined to modulate the polarity of the aqueous phase and interfacial contact angle for emulsification, enhance the NH_3_ solubility, and facilitate glycerol extraction.

## 2 Results and Discussion

As a *proof of concept*, we studied the ammmonolysis of glyceryl trilaurate (GTL) to lauramide under various conditions (**Table** **1**). First, a series of reactions were performed at 150 °C for 16 h with liquid ammonia at varying NH_3_:GTL ratios, with/without Aquivion PFSA (entries 1–5). Additional experiments were conducted in the presence of water (entries 6–8) and water/cosolvent mixtures (entries 9–13, entry 17) to assess the role of the interfacial surface area and *in‐situ* acidification by self‐assembled Aquivion PFSA particles on the GTL reactivity. Control experiments (entries 14–16) were carried out to isolate the catalytic impact of interfacial acidification.

**Table 1:** Emulsification and catalytic results for lauramide formation in different systems.a)

Entry	NH_3_/GTL [mol:mol]	PW98 eq [eq H^+^%]	Sonication	Emulsion[time = 0]	System	GTL conversion [%]	Yield lauramide [%]/NH_4_^+^ laurate [%]
1	47	–	No	No	–	11	9/1
2	47	0.64	No	No	–	<5	<1/<1
3	148	–	No	No	–	100	99/1
4^b)^	148	–	No	No	–	27	26/1
5^b)^	148	1.07	No	No	–	16	15/1
6^c)^	47	–	No	No	H_2_O	<1	‐/‐
7^c)^	47	0.64	No	No	H_2_O	<1	‐/‐
8^ d)^	47	0.64	Yes	Stable	H_2_O	33 ± 3	27 ± 2 /6 ± 1
9^e)^	47	–	No	Unstable	H_2_O/ACN (50:50)	37	35/2
10^e)^	47	–	Yes	Unstable	H_2_O/ACN (50:50)	100	63/37
11^e)^	47	0.64	Yes	Stable	H_2_O/ACN (50:50)	100	78 ± 4/22 ± 2
12^f)^	47	–	Yes	Unstable	H_2_O/EtOH (50:50)	100	35/16
13^f)^	47	0.64	Yes	Stable	H_2_O/EtOH (50:50)	100	72/8
14^e,g)^	47	0.64	No	Unstable	H_2_O/ACN (50:50)	100	60/40
15^e,g)^	47	0.64	Yes	Stable	H_2_O/ACN (50:50)	100	48/52
16^e,h)^	47	0.64	No	Unstable	H_2_O/ACN (50:50)	100	17/83
17^e,i)^	47	0.64	Yes	Stable	H_2_O/ACN (50:50)	100	73/27

a) Reaction conditions: 0.3 g GTL, 2.0 g NH_3_, 150 °C, 16 h. Other conditions.

b) 6 h.

c) First step: 0.90 mL H_2_O, 110 °C, 3 h; second step: 150 °C for 16 h.

d) Sonication; first step: 0.45 mL H_2_O, 110 °C, 10 h; second step: 150 °C for 16 h.

e) Addition of 0.45 mL water and 0.45 mL ACN; ^f)^Addition of 0.45 mL water and 0.45 mL EtOH.

g) Addition of 3 μmol NaOH (0.64 eq OH^−^% relative to GTL).

h) Lauric acid (0.29 g) was used instead of GTL.

i) Addition of Aquivion PW98 (3 mg, 0.64 eq H^+^% relative to GTL) combined with ammonium laurate (≈22 mg). The pressure was <10 bar and between 20 and 30 bar in all catalytic tests after 2.0 g and 3.0 g NH_3_ addition, respectively.

When only NH_3_ was introduced to the reactor (i.e. without water or Aquivion PW98, 47:1 NH_3_/GTL molar ratio in the active zone), the GTL conversion was only 11% with 9% and 1% yield of lauramide and ammonium laurate, respectively (entry 1). Traces of unidentified side products were detected, along with dilaurin and monolaurin intermediates (<1% yield). Addition of Aquivion PW98 (3 mg, 0.64 eq H^+^% relative to GTL) under the same reaction conditions reduced the GTL conversion to <5% (entry 2), indicating that the presence of acid inhibits the reaction.

To increase the conversion, the NH_3_/GTL molar ratio was raised to 148:1 (3.0 g NH_3_) which led to nearly complete conversion and a 99% lauramide yield (entry 3). Decreasing the reaction time from 16 h to 6 h under the same conditions reduced the GTL conversion to 27% with 26% and 1% yield of lauramide and ammonium laurate yield, respectively (entry 4). Adding Aquivion PW98 (3 mg, 1.07 eq H^+^% relative to GTL) under these conditions again suppressed the conversion to 16%, yielding only 15% lauramide (entry 5) matching the observation in entry 2. These results confirm that in non‐emulsified systems, NH_3_ is the key active catalytic species, and that large NH_3_ excess drives the reaction forward. However, the presence of acid Aquivion PW98 in suspension neutralizes NH_3_ and impedes the reaction.

To explore the role of emulsification, water (0.9 mL) was added to the system to enhance NH_3_ solubility and facilitate emulsion formation. The reaction was conducted in two steps: GTL hydrolysis at 110 °C for 3 h, followed by NH_3_ addition (1:47 GTL/NH_3_ molar ratio) and ammonolysis at 150 °C for 16 h. Without and with Aquivion PW98 (3 mg, 0.64 eq H^+^), GTL conversion remained negligible (<1%, entries 6 and 7). To promote emulsification, the system was sonicated with an ultra‐turrax, the water volume reduced to 0.45 mL, and the hydrolysis step extended to 10 h (entry 8). This generated a stable GTL‐in‐water emulsion (Figure S1, Supporting Information). Under these conditions, GTL conversion reached 33%, yielding 27% lauramide and 6% ammonium laurate, with full conversion of mono‐ and dilaurin intermediates. Notably, no lauric acid formed during the initial hydrolysis step, even with extended time, suggesting that lauramide formation occurred directly from GTL within the emulsion. Overall, these results suggest that GTL‐in‐water emulsions can activate direct GTL ammonolysis leading to the formation of lauramide and ammonium laurate without previous GTL hydrolysis to lauric acid.

**Figure 1:**
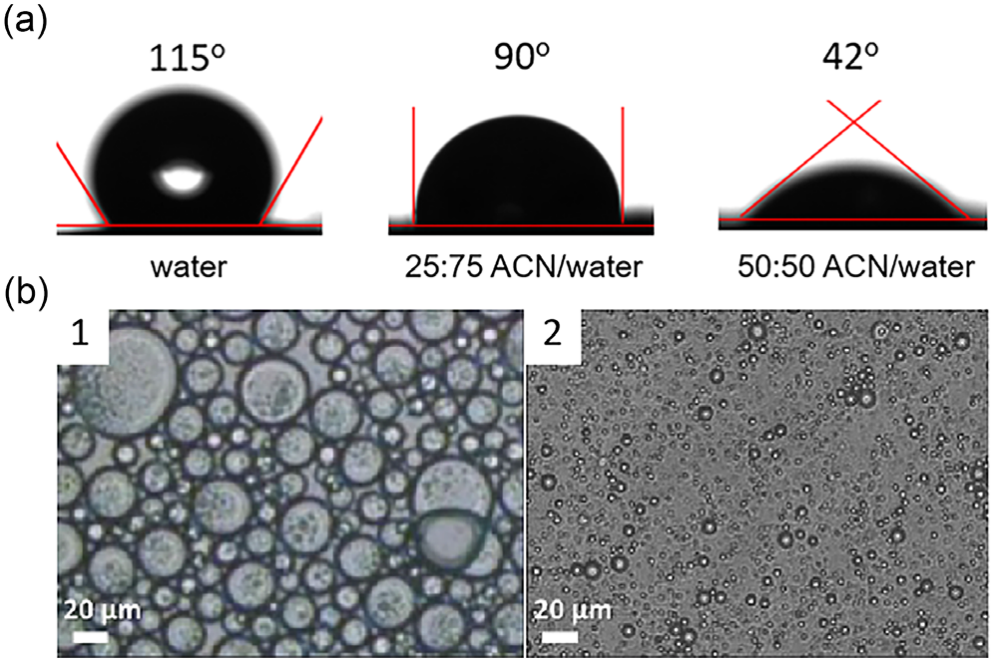
a) Contact angle measurements for pure water and ACN/water mixtures at variable ACN/water volume ratios. b) Optical images of GTL‐in‐water emulsions (b1) and GTL‐in‐water(ACN) emulsions (b2) stabilized by Aquivion PW98 particles at 50 °C after 3 days. Emulsification conditions: Aquivion PW98 was first dispersed in a 50:50 ACN/water (10 mg g^−1^) in a 20‐mL sealable tube. The dispersion was subjected to vigorous homogenization at 70 °C for 5 min (20,000 rpm), GTL (0.3 g) was added and a second homogenization step was conducted at the same conditions.

Based on these findings, water‐miscible co‐solvents were evaluated to further promote GTL ammonolysis. Acetonitrile (ACN) was selected first, known for lowering the energetic span of ammonolysis reactions.^[4c]^ ACN significantly reduced the air‐water surface tension from 72 mN/m (pure water) to 33 mN/m for 50:50 ACN/water mixtures (at 25 °C) (Table S1, Supporting Information), and lowered the contact angle on Aquivion PW98 pellets from 115° to 42° (**Figure** **1**a), improving wetting.

**Figure 2:**
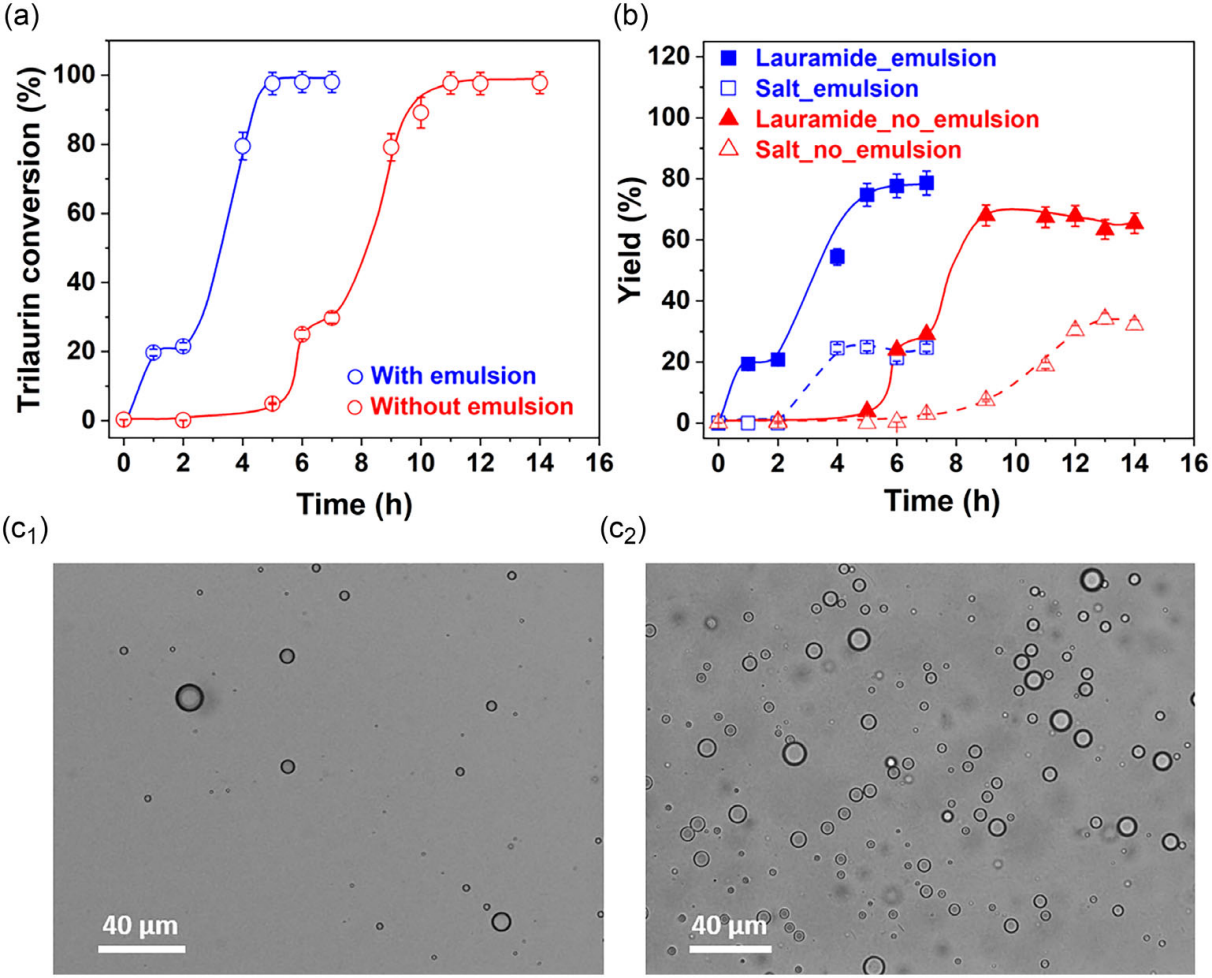
Time‐evolution of a) GTL conversion and b) lauramide and ammonium laurate yield (salt) with/without emulsion. Optical images of GTL‐in‐water(ACN) emulsions without sonication (no_emulsion) after stirring for c1) 5 min and c2) 4 h at 50 °C. Reaction conditions: 0.3 g GTL, 2.0 g NH_3_, 0.45 mL water, 0.45 mL ACN, 3 mg Aquivion PW98 (0.64 eq% with respect to GTL), and 150 °C. Emulsification was conducted by sonication, while for experiments without emulsion, Aquivion PW98 was added without sonication.

GTL‐in‐water emulsions (i.e., without ACN) had ≈50% dispersion with an average droplet size of 12.6 μm (Figure 1b1). Emulsions stabilized by Aquivion PW98 (3 mg, 0.64 eq H^+^% relative to GTL) with varying ACN/water ratios were analyzed by Turbiscan light scattering at 50 °C over 3 days (Figure S2, Supporting Information). At 25:75 ACN/water, emulsion stability and turbidity were similar to emulsions without ACN, with minor creaming effects in the bottom zone (Figure S2a, Supporting Information). At 50:50 ACN/water, dispersion improved to 80%–90% and average droplet size decreased to 3.2 μm (Figure 1b, 2), indicating significantly improved emulsion stability (Figure S2b, Supporting Information). Some clarification at the top and slight droplet growth (coalescence or flocculation) in the middle part of the emulsion was observed. Conversely, emulsions prepared with 75:25 ACN/water were unstable, likely due to excessive wettability of Aquivion PW98 particles (Figure S2c, Supporting Information), resulting in a very low contact angle (<42°) below measurable limits (Aquivion PW98 pellets were not stable during contact angle measurements). Based on these data, 50:50 ACN/water was chosen for further catalytic studies.

Next, we conducted the reaction at the same conditions as in Table 1 entry 1 (i.e. no emulsion), but using a 50:50 ACN/water mixture (entry 9). The GTL conversion increased to 37% with 35% lauramide yield. Ammonium laurate was also generated with 37% yield. Sonication improved conversion to 100%, yielding 63% lauramide and 37% ammonium laurate (entry 10), even if emulsions wereunstable. Notably, complete conversion was retained even when reducing the NH_3_ dosage from 2.0 g to 0.2 g (NH_3_/GTL molar ratio: 0.14:1) keeping the pressure in the range 2.4–10 bar, although the lauramide yield declined to 38% due to increased formation of ammonium laurate (Figure S3, Supporting Information).

To directly assess the effect of emulsification in the reactivity, we performed the reaction in GTL‐in‐water (ACN) emulsions (50:50 ACN/water) stabilized with Aquivion PW98 (3 mg, 0.64 eq H^+^% relative to GTL) were reacted under sonication (entry 11). Full GTL conversion was achieved, with lauramide and ammonium laurate yields of 78% and 22%, respectively. These results confirm that stable emulsions in the presence of Aquivion PW98 enhance lauramide formation over ammonium laurate.

To determine whether extracted glycerol influences the reaction, glycerol (GL) was added to the system from entry 11 at GL/GTL ratios of 1:1, 1:3, and 1:5. In all cases, lauramide yield remained unchanged, indicating that GL extraction does not significantly affect the catalytic outcome under these conditions.

Ethanol, another water‐miscible co‐solvent with low surface tension, was also evaluated (entries 12 and 13). Without Aquivion PW98, the lauramide yield was 35%, while it increased to 72% with Aquivion PW98 (3 mg, 0.64 eq H^+^% relative to GTL). Ammonium laurate yields were 16% and 8%, respectively. Ethyl laurate was detected as a byproduct. These results further support the role of emulsification in enhancing selectivity for amide formation.

With these results, we explored the catalytic role of Aquivion PW98 in GTL ammonolysis in GTL‐in‐water(ACN) emulsion. We measured the reactivity of the system at the same conditions as in entry 9 with Aquivion PW98 (3 mg, 0.64 eq H^+^% relative to GTL) but adding 3 μmol NaOH (1 eq H^+^) to fully neutralize Aquivion PW98 (entry 14). Full GTL conversion was achieved, with 60% lauramide and 40% ammonium laurate – results similar to the non‐Aquivion PW98 system under sonication (entry 10). When sonication was applied to this neutralized system (entry 15), conversion remained complete, but the lauramide yield dropped to 48% while ammonium laurate rose to 52%. These findings suggest that while emulsification increases overall conversion, interfacial acidification provided by Aquivion PW98 is crucial for lauramide selectivity. NH_4_^+^ ions likely act as catalytic species at the structured GTL‐water(ACN) interface.

To exclude the possibility that lauramide forms *via* dehydration of ammonium laurate, a control experiment was conducted using lauric acid (0.29 g) instead of GTL under entry 11 conditions with Aquivion PW98 (3 mg, 0.64 eq H^+^% relative to GTL) (entry 16). While lauric acid was fully consumed, lauramide yield dropped significantly – from 60% (without neutralization) to 17% (after neutralization) (see Supporting Information for details). Another test using Aquivion PW98 and ammonium laurate (entry 17) yielded 73% lauramide, comparable to 78% in entry 11, confirming that ammonium laurate did not interconvert to lauramide (or vice versa) under these conditions.^[^ ^17^ ^]^

Taken together, these results confirm the catalytic role of Aquivion PW98 in promoting direct GTL ammonolysis to lauramide. This is attributed to the enrichment of NH_4_^+^ ions at the GTL‐water(ACN) interface, where they act as active catalytic species. In contrast, added ammonium laurate together with Aquivion PW98 dissolves in the water/ACN phase without significant interfacial adsorption, thus keeping the related NH_4_^+^ cations far from the GTL‐water interface and not contributing to catalysis.

To further assess the catalytic role of Aquivion PW98 in lauramide synthesis, we monitored the kinetic profiles for GTL ammonolysis with/without emulsion induced by sonication (**Figure** **2**a,b). In the presence of emulsion (blue plots), the reaction was complete after 5 h, achieving a turnover frequency (TOF) of 31 h^−1^ for GTL conversion. In contrast, without emulsion (red plots), the reaction required 12 h to reach completion, displaying an induction period of *ca* 5 h and a much lower TOF of ≈4 h^−1^.

While higher yields of ammonium laurate were obtained without emulsion (80% vs. 60%), lauramide formation was consistently faster than ammonium laurate under both conditions. Notably, during the first 2 h in emulsion, only lauramide was formed followed by simultaneous formation of both lauramide and ammonium laurate. Both products reached a plateau after 4 h in emulsion. Without emulsion, the lauramide plateau was reached after 8 h, whereas ammonium laurate plateaued after 12 h. These results, along with the absence of a maximum in lauramide yield, confirm that ammonium laurate is not generated by lauramide hydrolysis, but rather by direct GTL hydrolysis in the presence of NH_3_.

Visual inspection revealed that the emulsion began to destabilize over the course of the reaction, transitioning to a single‐phase system after about 2 h – coinciding with complete lauramide formation (Figure 2b). During this initial period, a very high interfacial surface area (≈0.58 m^2^ for an average droplet size of 3.2 μm, Figure 1b2) was generated in emulsion, driven by the assembly of Aquivion PW98 particles at the GTL‐water (ACN) interface. To understand the slower reaction without emulsion (red plots), we examined the possible *in‐situ* formation of GTL droplets. After 5 min of stirring, few droplets were indeed observed, but after 4 h, droplet formation became more prominent, with an average size of 5.1 μm (Figure 2c1,c2), despite the system remaining visually biphasic and nonturbid. These findings emphasize the importance of interfacial acidification, which is accelerated by emulsification and facilitates the reaction, particularly during the early stage of the reaction (<2 h).

To explore the cause of emulsion destabilization after 2 h, we monitored the time‐ evolution of GTL‐in‐water (ACN) emulsions stabilized by Aquivion PW98 after NH_3_ addition and sonication. The emulsions remained stable for over 2 h but began coalescing after 20 h (Figure S4, Supporting Information). Zeta potential measurements of Aquivion PW98 suspensions in water revealed a consistent negative charge (–50 mV) irrespective of the pH (range from 7 to 10) (Figure S5, Supporting Information). Additionally, the zeta potential of GTL‐in‐water emulsions exhibited a negative surface charge of –38 mV, though with a broad distribution due to droplet polydispersity (Figure S6a, Supporting Information). Upon NH_3_ addition (≈30 wt% NH_4_OH) the zeta potential shifted from –38 mV to –72 mV, likely due to interfacial accumulation of OH^−^ anions (Figure S6b, Supporting Information). Similar trends were observed in emulsions stabilized by Aquivion PW98, although zeta potential distributions were narrower (Figure S6c,d, Supporting Information). In ACN/water mixtures, the zeta potential of Aquivion PW98 shifted from –1 mV to –15 mV upon NH_3_ addition, indicating lower ionization compared to pure water (Figure S7a,b, Supporting Information). A corresponding shift from –15 mV to –26 mV was observed in the stabilized emulsion (Figure S7c,d, Supporting Information). Collectively, these results suggest that NH_3_ alters the surface charge of Aquivion PW98 in water(ACN) dispersion, but the change in surface charge is not the primary driver of emulsion destabilization during the reaction.

To evaluate whether lauramide contributes to emulsion destabilization, we added lauramide to GTL‐in‐water(ACN) emulsions at a lauramide/GTL molar ratio of 1:3. Emulsion stability dropped sharply – from over 5 h to under 10 min (Figure S8, Supporting Information). Lauramide was found to dissolve into the oil phase without precipitating, suggesting it interferes with Aquivion PW98 adsorption at the interface, though the reaction still proceeded to full GTL conversion within 5 h (Figure 2).

**Figure 3:**
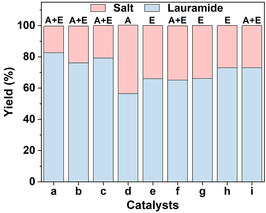
Yield of lauramide and ammonium laurate (salt) for the GTL‐water (ACN) system in the presence of variable loading of Aquivion PW98 loading a) 1.5 mg, b) 3.0 mg, c) 6.0 mg, d) 3 μmol PTSA, e) 3 mg silica‐F, f) 3 μmol PTSA and 3 mg silica‐F mixture, g) 3 mg silica‐C8 particles, h) the mixture of 3 mg Aquivion PW98 and 22 mg ammonium laurate, and i) second run of b). Reaction conditions: 0.3 g GTL, 2.0 g NH_3_, 0.45 mL water, 0.45 mL ACN, 150 °C, and 8 h. The systems were sonicated before reaction. Nomenclature: A = acid, E = emulsion, A/E = acid particle (Aquivion PW98) and A + E = Acid (homogeneous) + Emulsion.

To further elucidate the role of interfacial acidification, we conducted control experiments with various acids. First, we varied Aquivion PW98 loading (0.64–2.6 eq% or 0.5–2.0 wt% relative to GTL) in GTL‐in‐water (ACN) emulsions (**Figure** **3**a–c). Lauramide yields remained consistently high (75%–82%), indicating that even low catalyst loadings (0.5 wt%) are sufficient to drive interfacial acidification and promote direct GTL ammonolysis. In contrast, replacing Aquivion PW98 with homogeneous p‐toluenesulfonic acid (PTSA, 0.64 eq%) under non‐emulsified conditions resulted in a reduced lauramide yield (56%) despite full GTL conversion (Figure 3d), highlighting the importance of surface activity for catalytic performance.

We also tested non‐acidic, surface‐active silicas functionalized with perfluorodecyltrimethoxysilane (silica‐F) and octyl groups (silica‐C8) (Figure 3e,g; Table S2, Supporting Information). In both cases, stable emulsions were formed, yet lauramide yields declined to 65%, underscoring the necessity of interfacial acidification, not just emulsification. Combining silica‐F with PTSA did not improve the performance (Figure 3f), further confirming the key role of interfacial acidification by NH_4_^+^ induced by Aquivion PW98 adsorbed at the GTL/water(ACN) interface in enhancing GTL ammonolysis to lauramide.

A recycling test was performed by recovering the solid residue (comprising ammonium laurate and Aquivion PW98) after product separation and reusing it with fresh GTL (0.3 g) under identical sonication conditions (see Supporting Information for details; Figure S9). The lauramide yield remained high (73%), comparable to the initial reaction (78%, Table 1, entry 11; Figure 3i). Also, zeta potential and dynamic light scattering (DLS) measurements of recycled Aquivion PW98 exhibited no significant changes after the reaction (Figure S10, Supporting Information). These results demonstrate the durability and reusability of Aquivion PW98 under reaction conditions.

## 3 Conclusions

In summary, Aquivion PW98, formulated as acidic powder (1.02 mmol H^+^/g), effectively catalyzes the direct ammonolysis of GTL to lauramide in GTL‐water (acetonitrile) emulsions at relatively mild conditions (150 °C) and significantly reduces the requirement of ammonia excess compared to conventional ammonolysis with liquid NH_3_. Formation of sub‐3.2 μm emulsion droplets greatly increases interfacial area and promotes interfacial acidification via NH_4_^+^ enrichment, facilitating “on‐water” catalysis. Under these optimized conditions, lauramide formation is enhanced sevenfold at low ammonia excess (47:1 mol/mol NH_3_/GTL) and its yield is nine times higher after 16 h than when using liquid ammonia. Emulsion destabilization due to lauramide formation simplifies product separation and facilitates catalyst recycling, enhancing both the economic and environmental sustainability of the process.

## 4 Experimental Section

The details of the experiments are provided in the Supporting Information.

## Conflict of Interest

The authors declare no conflict of interest.

## Supporting information

Supplementary Material
