# Supplementary material for: “On‐Water” Interfacial Acidification Enhances Direct Ammonolysis of Triglycerides
Source: ChemSusChem. 2025 Aug 12;18(19):e202500912. doi: 10.1002/cssc.202500912 (PMC12487732; doi:10.1002/cssc.202500912)
Supplement: Supplementary file 1 — Supplementary Material [file CSSC-18-e202500912-s001.pdf]

'On-Water' Interfacial Acidification Enhances Direct Ammonolysis of Triglycerides

Jie Gao,[a]† Kang Wang,[c]† Yunjiao Gu[b] and Marc Pera-Titus[a,c]*

[a] Eco-Efficient Products and Processes Laboratory (E2P2L), UMI 3464 CNRS-Solvay, 3966 Jin Du Road, Xin Zhuang Ind. Zone, 201108 Shanghai, China

[b] Solvay China (Co), 3966 Jin Du Road, Xin Zhuang Ind. Zone, 201108 Shanghai, China

[c] Cardiff School of Chemistry, Cardiff Catalysis Institute, Translational Research Hub, Cardiff University, Maindy Road, Cardiff, CF24 4HQ

* Corresponding author. E-mail: peratitusm@cardiff.ac.uk

ELECTRONIC SUPPORTING INFORMATION

Figure S1. The microscopic images of the emulsions (a) before and (b) after hydrolysis. Conditions: 3 mg Aquivion® PW-98, 0.3 g GTL, 0.90 mL H2O, 110 °C, 3 h, sonication.

Figure S2. Dynamic light scattering spectra measured in Turbiscan for GTL-in-water(ACN) emulsions prepared at variable water/ACN volume ratios after stabilization at 50 °C for 3 days.

Figure S3. Evolution of the lauramide and ammonium laurate yields with the initial amount of NH_3 loaded to the autoclave. Reaction conditions: 0.3 g GTL, 2.0 g different weights of NH_3, 0.45 mL water, 0.45 mL acetonitrile, 150 °C, 16 h, sonication before the reaction.

Figure S4. Time-evolution of GTL/water(ACN) emulsions: (a) 0 h, (b) 2 h, (c) 20 h. Emulsification conditions: 3 mg Aquivion® PW98 (0.64 eq% with respect to GTL) 0.3 g GTL, 0.45 mL water, 0.45 mL ACN, 50 °C, 2 mL ammonia water sonication for 5 min at 20,000 rpm.

Figure S5. Zeta potential of Aquivion® PW98 dispersion in (a) water and (b) aqueous NH_3 solution. The dispersion was prepared by adding 5 mg Aquivion® PW98 in 5 mL water and 5 mg dispersed in Aquivion® PW98 in 5 mL of 0.5wt% aqueous NH_3 solution.

Figure S6. Zeta potential of (a) GTL-in-water emulsions stabilized after sonication, (b) GTL-in-NH_4OH emulsions stabilized after sonication, (c) GTL-in-water stabilized by Aquivion® PW98 followed by sonication, and (d) GTL-in-NH_4OH emulsion stabilized by Aquivion® PW98 followed by sonication. Emulsification conditions: 0.05 g GTL, 2 mL water or 0.5 wt% aqueous NH_3 solution, with/without 2 mg Aquivion® PW98, sonication at 20,000 rpm for 5 min.

Figure S7. Zeta potential of Aquivion® PW98 dispersion in (a) water(ACN) and (b) NH_4OH(0.5wt%)(ACN) solution. The dispersion was prepared by adding 5 mg Aquivion® PW98 in 5 mL water(ACN) or NH_4OH solution and ACN, (c) GTL-in-water(ACN) and (d) GTL-in-NH_4OH(ACN) emulsions stabilized by Aquivion® PW98 followed by sonication, (Emulsification conditions: 0.05 g GTL, 1 mL water or 0.5 wt% aqueous NH_3 solution, 1 mL NH_4OH solution, 2 mg Aquivion® PW98, sonication at 20,000 rpm for 5 min.

Figure S8. Time-evolution of GTL/water(ACN) emulsions stabilized by Aquivion® PW98 without and with lauramide, respectively. Experimental conditions: oil phase: 0.3 or 0.2 g GTL / 0.1 g lauramide; aqueous phase: 0.9 mL water or 0.45 mL water / 0.45 mL ACN, 3 mg Aquivion® PW 98, sonication at 20,000 rpm for 5 min.

Figure S9. Optical images of the reaction mixture after reaction (a) at 100 °C and (b) at room temperature. Reaction conditions: 0.3 g GTL, 2.0 g NH_3, 0.45 mL water, 0.45 mL ACN, 150 °C, 8 h, sonication before the reaction.

Figure S10. (a) Zeta potential and (b) dynamic light scattering measurements of fresh and spent Aquivion® PW98 dispersion in water. The dispersion was prepared by adding 5 mg fresh or spent Aquivion® PW98 in 5 mL of water.

Table S1. Surface tensions of water/ACN mixtures with different compositions and GTL at 80 °C.

Table S2. Main properties of silica particles.[1]

Experimental

Reagents and materials

Tetraethyl orthosilicate (TEOS, 98%), 1H,1H,2H,2H-perfluorodecyltriethoxysilane (PFDTES, 97%), triethoxy(octyl)silane (90%), (3-mercaptopropyl)triethoxysilane (MPTES, 97%), ammonium hydroxide solution (28-30%) and anhydrous ethanol (99.9%), all purchased from Sigma-Aldrich, were used for the synthesis of silica particles. Aquivion® PFSA (powder form, PW98, 1.02 mmolH^+/g) was supplied by Solvay Special Chemicals. Glyceryl trilaurate (GTL, 99.5%), provided by Solvay, was used as model vegetable oil. P-toluenesulfonic acid monohydrate (PTSA, 98.5%, TCI-EP) was used as homogeneous catalysts. Acetone (>99.5%, Sigma-Aldrich) and biphenyl (>99%) were used for conducting the recycling tests and as internal standard, respectively. All the chemicals were used as received without further purification.

Preparation of alkyl-functionalized particles

The C8-functionalized silica particles (silica-C8) were prepared by the Stöber method using an established protocol.[S1] In a typical synthesis, tetraethyl orthosilicate (TEOS, 3 mL) was rapidly added into a mixture of ethanol (37 mL), deionized water (1.6 mL), and an NH_3 aqueous solution (25-28%, 3.2 mL). Then, triethoxy(octyl)silane (1 mL) and 3-mercaptopropyl triethoxysilane (MPTES, 0.3 mL) were added to the solution. After mixing for 2 h, the resulting particles were collected by centrifugation, washed three times with ethanol, and dried at 80 °C overnight.

The fluorinated silica particles (silica-F) were also prepared by the Stöber method.[S1] In a typical synthesis, TEOS (4 mL), deionized water (11.2 mL) and an $NH_3{\cdot}H_2O$ (1.6 mL) were dissolved in ethanol (80 mL) at 40 °C. Then, a mixture of 1H,1H,2H,2H-perfluorodecyltriethoxysilane (PFDTES, 1.97 mL) and MPTES (0.27 mL) was added under fast stirring, and the solution was reacted for 30 min. The particles were separated by centrifugation, washed three times with ethanol, and dried at 80 °C overnight.

The main properties of both particles are listed in the **Table S2**.

Emulsification studies

Preparation of emulsions: The GTL-in-water and GTL-in-water(acetonitrile) emulsions were prepared according to the following method. First, the given solid emulsifier (e.g., PW98) was dispersed in water or water/acetonitrile mixtures (0.45 mL or 0.9 mL total volume, 10 mg/g) in a 20-mL sealable tube. The dispersion was the subjected to vigorous homogenization at 70 °C for 5 min (20,000 rpm, Ultraturrax Fluko FA25, 8 mm dispersing tool). Subsequently, GTL (0.3 g) was added and a second homogenization step was conducted at the same conditions. After preparation, the emulsified system was kept at 50 °C for 3 days to assess the stability of the emulsified phase.

Emulsion volume and droplet size: The emulsion volume was determined by direct inspection of the emulsions using a Nikon D300s camera equipped with a macro lens (AF-S Macro Nikon 105 mm 1:2.8G ED) and NK remote software. The continuous phase was appraised using the dilution

method by dropping one droplet of the emulsion into water and GTL separately. The droplet size was measured with an Olympus IX-51 light transmission microscope equipped with x10 ocular, x4, x10, x40 and x100 objectives and DP2-BSM software. Visilog software was used to analyze the droplet size. The air-water(acetonitrile) surface tension was measured on a Sigma 700 tensiometer (Biolin Scientific AB) equipped with a Wilhelmy plate and a Du Noüy ring set at 3 mm/min for low interfacial tensions. The emulsions were also characterized by dynamic light scattering using a Turbiscan apparatus for the whole height of the emulsions at variable volume percentages of acetonitrile in water.

Contact angle measurements: The contact angles were determined using the sessile drop method with a Dataphysics OCA 35 device. A 5 μL liquid droplet was deposited on pellets made from powder, with each measurement repeated three times per sample. The pellets were prepared by compressing at least 200 mg of particle powder under a 5-ton load for 5 minutes. The shape of the droplets was analyzed to measure the contact angle.

Zeta potential measurements: The zeta potential of Aquivion® PW98 dispersion in water was measured using a Malvern Zetasizer Nano ZS zeta potential analyzer at 25 °C. Around 800 μL of well-dispersed Aquivion® PW98 dispersion were transferred into a Malvern Folded Capillary Cell DTS1070. Each measurement was then passed in 100 runs. The repeatability for each dispersion was confirmed after 3 measurements.

Catalytic tests

In a typical experiment, GTL (0.3 g, 0.47 mmol), water (0.45 mL), acetonitrile (0.45 mL) and a given amount of catalyst were added into a stainless-steel autoclave (Taiatsu, 40 mL) and homogenized according to the emulsification protocol described above. A given weight of NH_3 was then added to the autoclave using a water/ice bath. The autoclave was then insulated with an alumina jacket, and the mixture was heated to 150 °C using an IKA® C-MAG-HS hotplate and stirred for 16 h at 750 rpm. The nominal NH_3/GTL ratio in these experiments was 250:1 with a NH_3/GTL ratio of 47 in the liquid zone. Ammonolysis tests with pure NH_3 (3.0 g) were carried out at the same reaction conditions, but without adding water and acetonitrile to the autoclave. The nominal NH_3/GTL ratio was 375:1 with a NH_3/GTL ratio of 148 in the liquid zone.

After the reaction, the mixture was cooled down to room temperature and NH_3 was released until ambient pressure was reached. Acetone (20 mL), together with biphenyl (10-20 mg) as internal standard, was added to dissolve lauramide and form one single phase. Then, the catalyst was separated using a disposable syringe filter with 220-nm pore size. The solution was analyzed by gas chromatography using an Agilent 7890 GC equipped with a FID detector and a HT-5 capillary column (length 30 m, i.d. 0.25 mm, film thickness 0.25 mm). The different products were identified by GC-MS (Agilent 6890/5973).

The GTL conversion, lauramide and ammonium laurate yields and turnover frequency (TOF) for GTL conversion were calculated by interpolation of the corresponding calibration curves using biphenyl as internal standard as follows

$$\mathrm{GTL\ conversion(t)} = 1 - \frac{\mathrm{n_{GTL}(t)}}{\mathrm{n_{GTL}^{0}}}\ \mathrm{x100} \qquad \text{(S1)}$$

$$\mathrm{Lauramide\ yield(t)} = \frac{\mathrm{n_{Lauramide}(t)}}{\mathrm{n_{GTL}^{0}}}\ \mathrm{x100} \qquad \text{(S2)}$$

$$\mathrm{Ammonium\ laurate\ yield(t)} = \frac{\mathrm{n_{Ammonium\ laurate}(t)}}{\mathrm{n_{GTL}^{0}}}\ \mathrm{x100} \qquad \text{(S3)}$$

$$\mathrm{TOF(t=0)} = \frac{1}{n_{H^+}} \frac{d\mathrm{n_{GTL}(t)}}{dt} \qquad \text{(S4)}$$

where $\mathrm{n_{GTL}^{0}}$ and $\mathrm{n_{GTL}^{0}(t)}$ refer to the mole number of GTL at time = 0 and time = t, respectively, and $n_{Lauramide}$(t) and $n_{Ammonium\ laurate}$ (t) are the mole numbers of lauramide and ammonium laurate, respectively, at time = t.

The catalytic tests were repeated at least three times to ensure reproducibility.

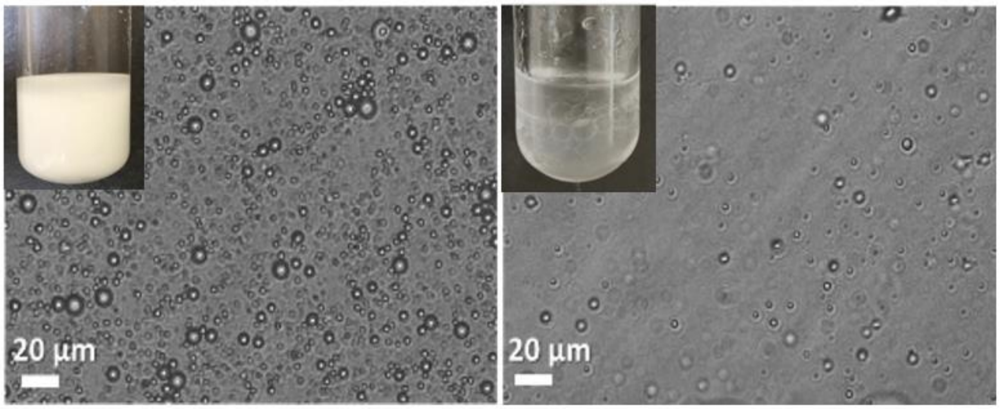

Figure S1. Optical images of emulsions for the GTL/water system (a) before and (b) after hydrolysis. Conditions: 3 mg Aquivion® PW-98, 0.3 g GTL, 0.90 mL H_2O, 110 °C, 3 h, sonication.

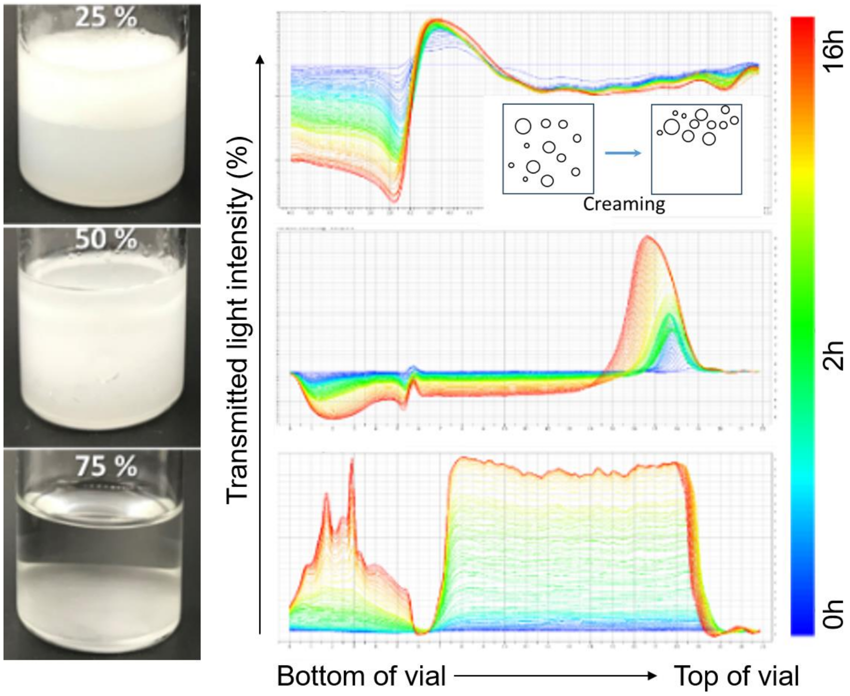

Figure S2. Dynamic light scattering spectra measured in a Turbiscan for GTL-in-water(ACN) emulsions prepared at variable Water/ACN volume ratios after stabilization at 50 °C for 3 days.

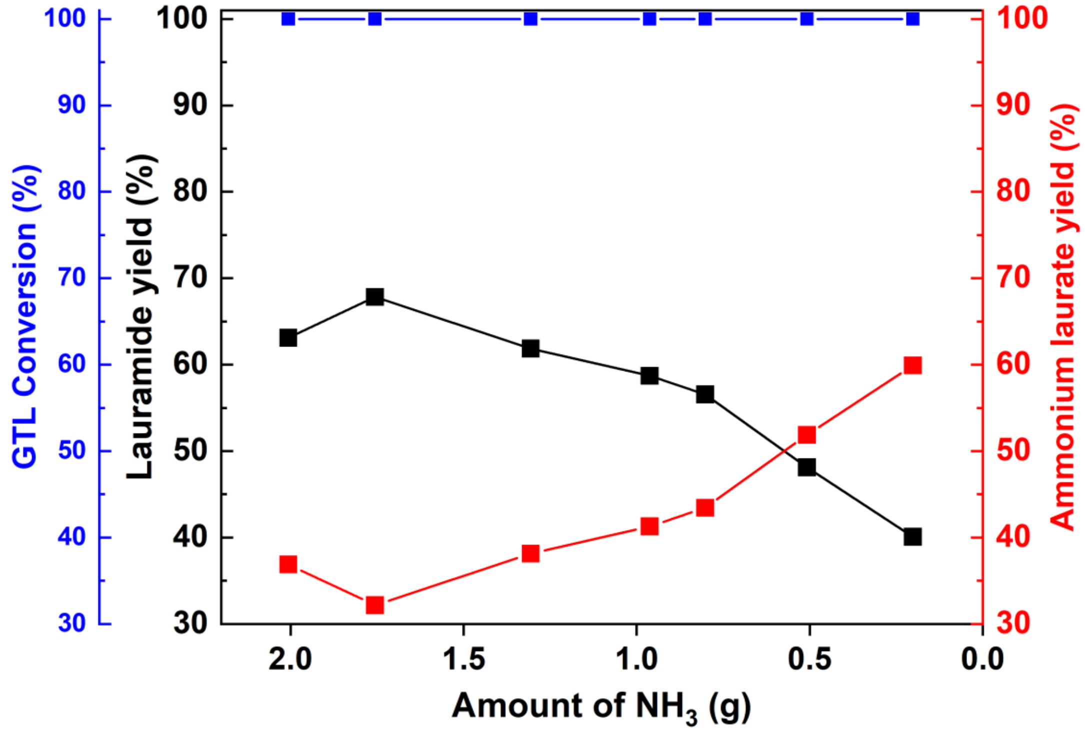

Figure S3. Evolution of GTL conversion and lauramide and ammonium laurate yields as a function of the initial amount of NH_3 loaded to the autoclave. Reaction conditions: 0.3 g GTL, 0.45 mL water, 0.45 mL ACN, 150 °C, 16 h, sonication before the reaction.

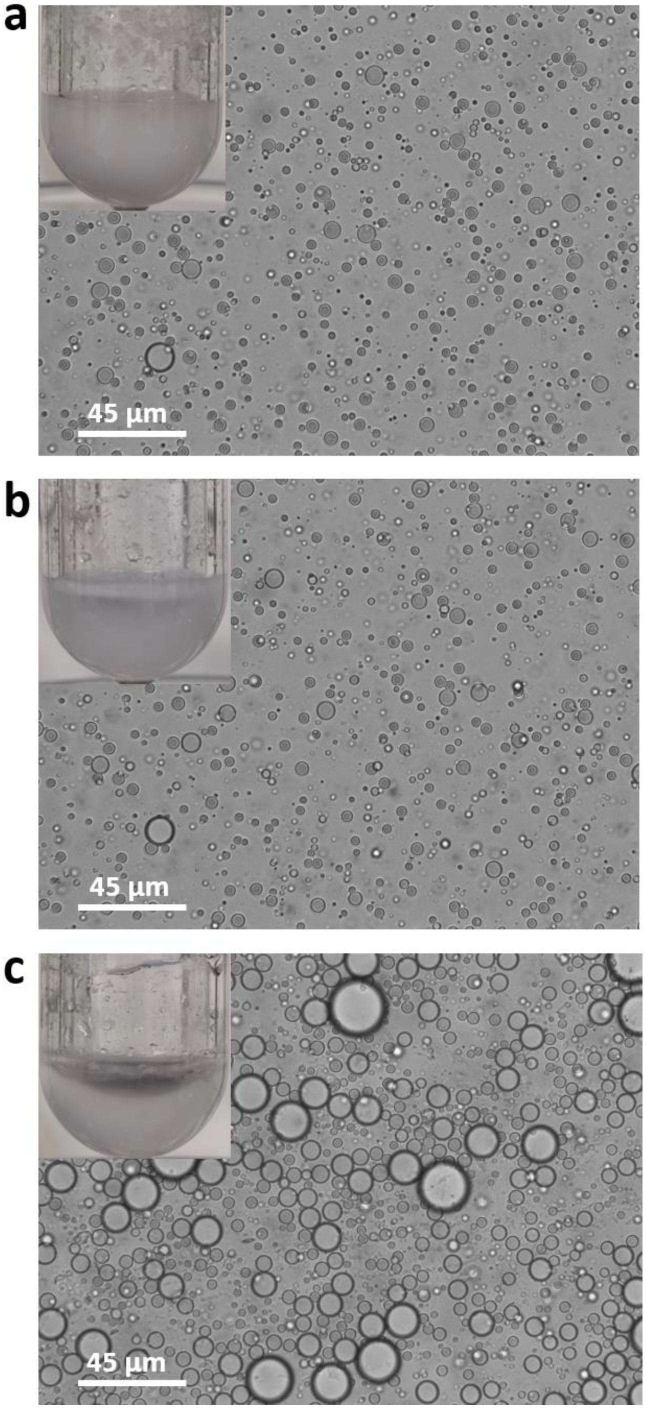

Figure S4. Time-evolution of GTL/water(ACN) emulsions: (a) 0 h, (b) 2 h, (c) 20 h. Emulsification conditions: 3 mg Aquivion® PW98 (0.64 eq% with respect to GTL), 0.3 g GTL, 0.45 mL water, 0.45 mL ACN, 50 °C, 2 mL ammonia water sonication for 5 min at 20,000 rpm.

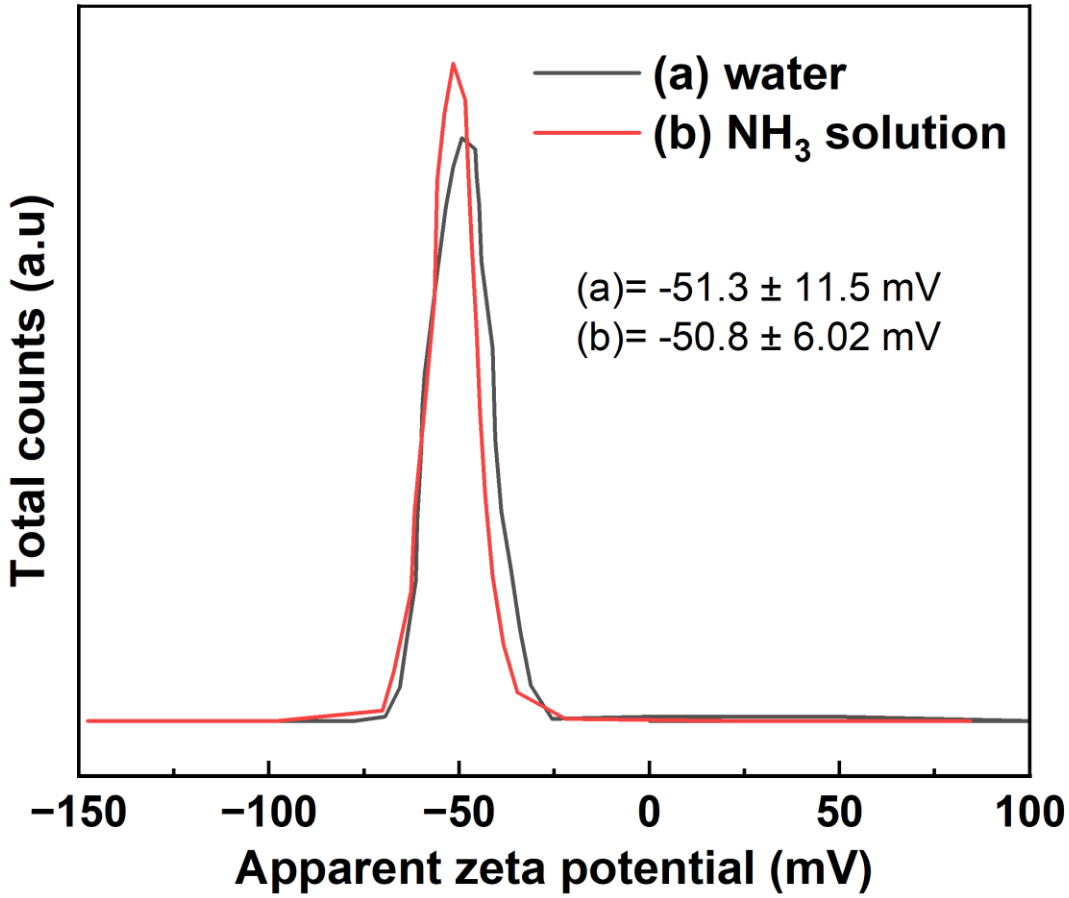

Figure S5. Zeta potential of Aquivion® PW98 dispersion in (a) water and (b) aqueous NH_3 solution (0.5wt%). The dispersion was prepared by adding 5 mg Aquivion® PW98 in 5 mL water or water containing NH_3.

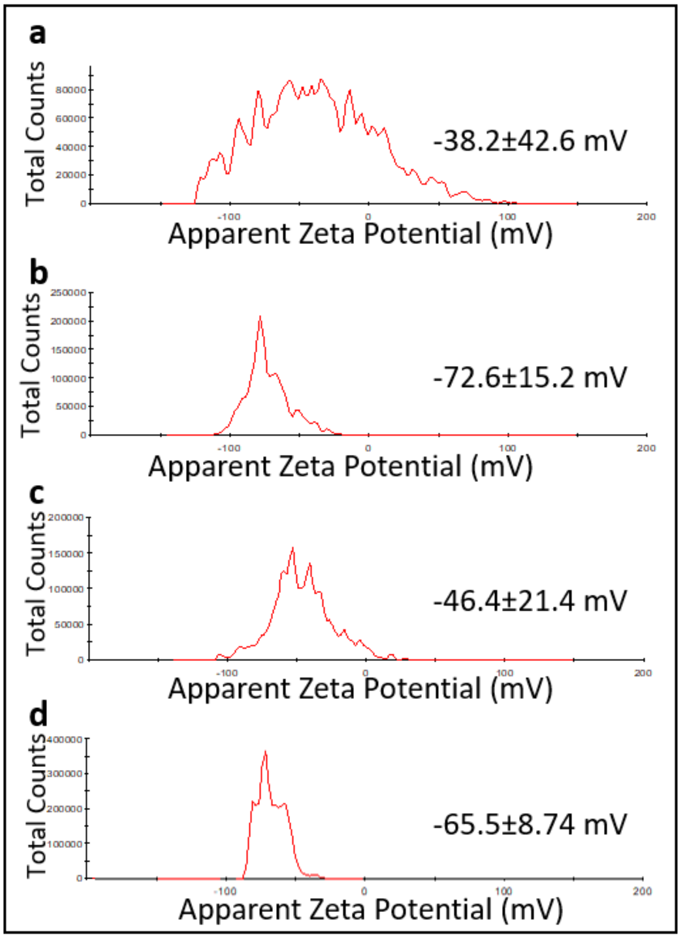

Figure S6. Zeta potential of (a) GTL-in-water emulsions stabilized after sonication, (b) GTL-in-NH_4OH emulsions stabilized after sonication, (c) GTL-in-water stabilized by Aquivion® PW98 followed by sonication, and (d) GTL-in-NH_4OH emulsion stabilized by Aquivion® PW98 followed by sonication. Emulsification conditions: 0.05 g GTL, 2 mL water or 0.5 wt% aqueous NH_3 solution, with/without 2 mg Aquivion® PW98, sonication at 20,000 rpm for 5 min.

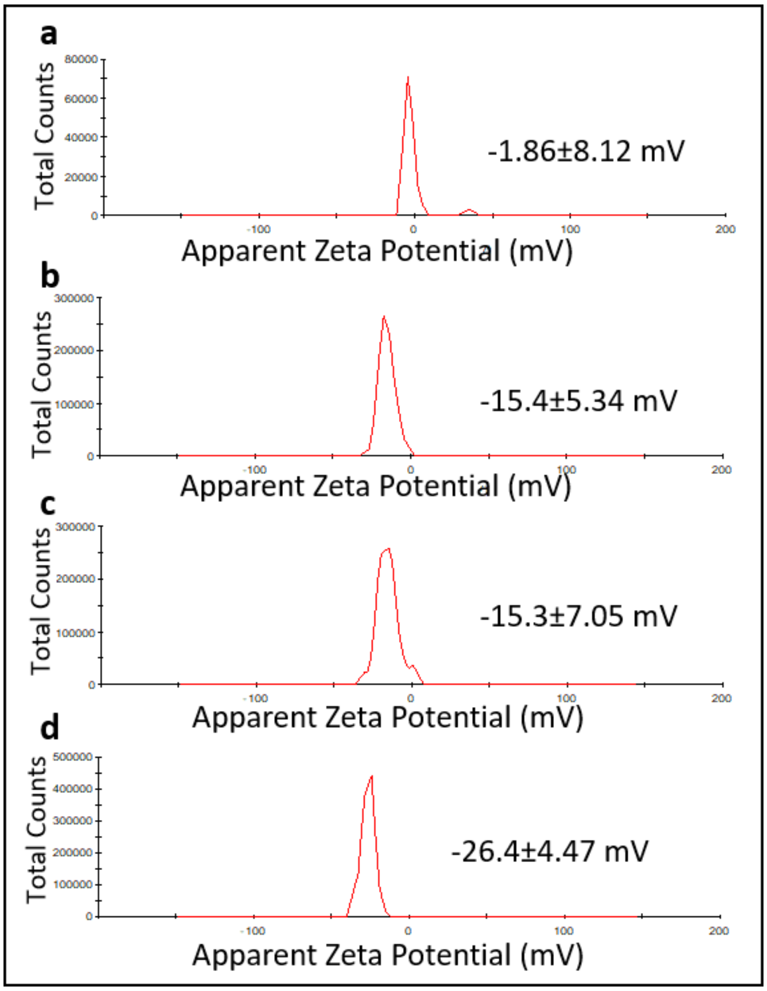

Figure S7. Zeta potential of Aquivion® PW98 dispersion in (a) water(ACN) and (b) NH_4OH(0.5wt%)(ACN) solution. The dispersion was prepared by adding 5 mg Aquivion® PW98 in 5 mL water(ACN) or NH_4OH solution and ACN, (c) GTL-in-water(ACN) and (d) GTL-in-NH_4OH(ACN) emulsions stabilized by Aquivion® PW98 followed by sonication, (Emulsification conditions: 0.05 g GTL, 1 mL water or 0.5 wt% aqueous NH_3 solution, 1 mL NH_4OH solution, 2 mg Aquivion® PW98, sonication at 20,000 rpm for 5 min.

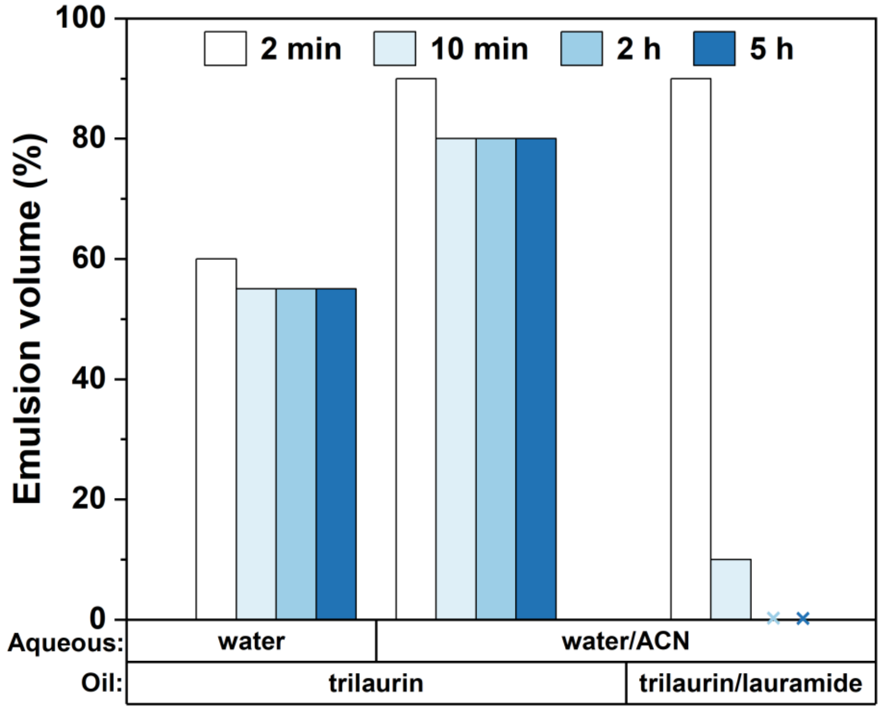

Figure S8. Time-evolution of GTL/water(ACN) emulsions stabilized by Aquivion® PW98 without and with lauramide, respectively . Experimental conditions: oil phase: 0.3 or 0.2 g GTL / 0.1 g lauramide; aqueous phase: 0.9 mL water or 0.45 mL water / 0.45 mL ACN, 3 mg Aquivion® PW 98, sonication at 20,000 rpm for 5 min.

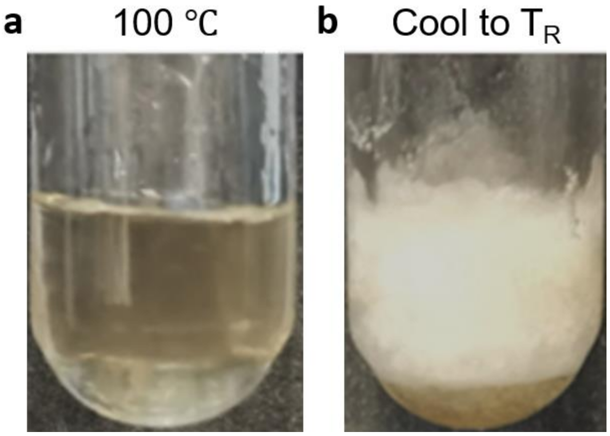

Figure S9. Optical images of the reaction mixture after reaction (a) at 100 °C and (b) at room temperature. Reaction conditions: 0.3 g GTL, 2.0 g NH_3, 0.45 mL water, 0.45 mL ACN, 150 °C, 8 h, sonication before the reaction.

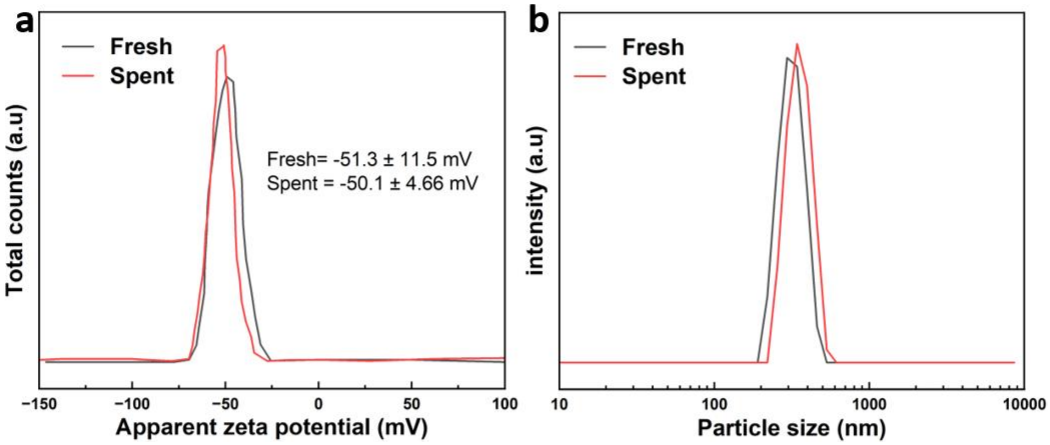

Figure S10. (a) Zeta potential and (b) dynamic light scattering measurements of fresh and spent Aquivion® PW98 dispersion in water. The dispersion was prepared by adding 5 mg fresh or spent Aquivion® PW98 in 5 mL of water.

Table S1. Surface tensions of water/ACN mixtures with different compositions and GTL at 80 °C

Liquid	Surface tension mN/m
H_2O	72.14±0.06
25% CH_3CN in H_2O	39.65 ±0.08
37.5% CH_3CN in H_2O	34.98±0.05
50% CH_3CN in H_2O	33.12±0.06
75% CH_3CN in H_2O	31.78±0.04
CH_3CN	29.22±0.04
Trilaurin-80 °C	28.4

Table S2. Main properties of silica particles.[1]

Particles	Weight loss (wt%)[a]	F (wt%)[a]	$\bar{D}_{Silica}$ (nm)[b]
Silica-F	53	33	364
Silica-C8	12	-	244

[a] Measured by TGA; [b] Measured by HR-TEM

References:

S1. Zhang, S., et al., Pickering interfacial catalysis for aerobic alcohol oxidation in oil foams. *Journal of the American Chemical Society*, 2022. **144**(4): p. 1729-1738.
